# The role of NSE/GCS ratio on the seventh day as an independent predictor of 90-day outcomes in patients with severe traumatic brain injury

**DOI:** 10.5937/jomb0-61005

**Published:** 2026-02-27

**Authors:** Chunying Zhu, Xiaona Liu, Huan Wang, Yingfu Zhang, Wei Li

**Affiliations:** 1 The First Central Hospital of Baoding, Department of Neuroscience Intensive Care Unit, Baoding, Hebei Province, China; 2 Lai Shui County Hospital of Baoding, Department of Critical Care Medicine, Baoding, Hebei Province, China; 3 The First Central Hospital of Baoding, Endoscopic Diagnosis and Treatment Centre, Baoding, China

**Keywords:** neuron-specific enolase, Glasgow Coma Scale, biomarker, prognosis, traumatic brain injury, neuronski specifična enolaza, Glazgov koma skala, biomarkeri, prognoza, traumatska povreda mozga

## Abstract

**Background:**

Traumatic brain injury (TBI) can lead to secondary damage that affects patient prognosis. Neuron-specific enolase (NSE) in the blood serves as a marker of nerve damage, while the Glasgow Coma Scale (GCS) score indicates the level of consciousness in patients. The ratio of NSE to GCS (NGR) during the perioperative period can help assess the 90-day prognosis in patients with severe TBI.

**Methods:**

This study involved 63 severe TBI patients. We collected their clinical and preadmission lab data. After admission, we provided personalised, comprehensive treatment, including blood tests for interleukin 6 (IL-6), NSE, blood osmotic pressure (OSM), procalcitonin (PCT), and D-dimer (DD). We evaluated the GCS score on Day 7 to calculate NGR. Using logistic regression and the receiver operating characteristic (ROC) curve, we analysed Preadmission NLR0, NSE7, GCS7, and NGR7 on the seventh day of admission.

**Results:**

Patients with poor 90-day outcomes were older, had more extended hospital stays, and exhibited higher levels of NLR0 and IL-6 upon admission (all p&lt;0.05). NLR0, NSE7, GCS7 scores, and NGR7 independently predicted poor outcomes in severe TBI patients. NGR7 showed a strong Pearson r value (r=-0.702, p&lt;0.0001) and the highest diagnostic accuracy for 90-day prognosis Šarea under the curve (AUC) = 0.932; 95% CI =0.872-0.993.

**Conclusions:**

NGR on the seventh day post-admission significantly predicts 90-day outcomes in severe TBI patients.

## Abstract (sr)

**Uvod:**

Traumatska povreda mozga (TPM) često dovodi do sekundarnih ostećenja koja značajno utiču na ishod lečenja. Neuronski specifična enolaza (NSE) u serumu predstavlja marker oštećenja nervnog tkiva, dok Glazgov koma skala (GCS) odražava stepen poremećaja svesti. Odnos NSE i GCS (NGR) u perioperativnom periodu može imati prognostički značaj za 90-dnevni ishod kod bolesnika sa teškom TPM. M etode: U studiju je uključeno 63 bolesnika sa teškom TPM. Analizirani su klinički podaci i laboratorijski nalazi pre prijema. Nakon hospitalizacije, primenjen je individualizovan i sveobuhvatan terapijski pristup, uključujući određivanje interleukina 6 (IL-6), NSE, osmotskog pritiska (OSM), prokalcitonina (PCT) i D-dimera (DD). Sedmog dana je procenjen GCS i izračunat NGR. Logističkom regresionom analizom i ROC krivom su ispitane prognostičke vrednosti parametara NLR0, NSE7, GCS7 i NGR7.

**Rezultati:**

Bolesnici sa nepovoljnim 90-dnevnim ishodom bili su stariji, duže hospitalizovani i imali više vrednosti NLR0 i IL-6 pri prijemu (svi p&lt;0,05). NLR0, NSE7, GCS7 i NGR7 pokazali su se kao nezavisni prediktori nepovoljnog ishoda. NGR7 je imao najjaču Pearsonovu korelaciju (r=-0,702; p&lt;0,0001) i najveću dijagnostičku tačnost u predviđanju 90-dnevnog ishoda [AUC = 0,932; 95% CI = 0,872-0,993; granična vrednost =4,69; senzitivnost = 86,49%; specifičnost =92,31%].

**Zaključak:**

NGR sedmog dana nakon prijema predstavlja značajan nezavisni prediktor 90-dnevnog ishoda kod bolesnika sa teškom TPM.

## Introduction

Severe traumatic brain injury stands as a frequent critical neurological ailment encountered in clinical practice. The principal harm primarily arises from occurrences such as traffic accidents, falls, and mishaps, resulting in a range of dysfunctions. The ailment exhibits variability and swift progression, often accompanied by disruptions in consciousness. Subsequent injury poses a significant peril to patients' lives, boasting elevated rates of both disability and fatality. Secondary injury represents a critical point for clinical intervention in traumatic brain injury, significantly influencing patient prognosis. Neuron-specific enolase (NSE) primarily resides within neurons and neuroendocrine cells. Following a traumatic brain injury, the blood-brain barrier is compromised, leading to neuronal damage and subsequent release of NSE into the peripheral bloodstream. This results in a substantial increase in serum NSE levels [1][2]. Excessive inflammatory responses can further exacerbate secondary damage post-traumatic brain injury (TBI). The imbalance between pro-inflammatory and anti-inflammatory systems has a significant impact on patient prognosis. The Glasgow Coma Scale (GCS) is a paramount neurological tool for assessing neurological function in patients with traumatic brain injury, serving as a crucial gauge for consciousness levels and disease severity. While NSE and GCS, independently or in tandem, bear prognostic utility for traumatic brain injury, their association with 90-day outcomes remains underexplored [3]. In this study, we scrutinised the 90-day prognosis of severe TBI patients, leveraging perioperative clinical indicators linked to severe cranio-cerebral trauma, along with serum NSE levels and GCS scores taken on the 7th day post-admission. The prognostic value of NSE and GCS has been extensively studied in various neurological conditions, particularly TBI and stroke. NSE, a biomarker indicative of neuronal damage, has been shown to correlate with the severity of brain injuries, with elevated levels associated with worse outcomes and increased mortality rates. In stroke patients, higher NSE levels post-event are linked to poor prognoses, reflecting the extent of neuronal injury.

Meanwhile, GCS serves as a critical clinical tool for assessing consciousness, with lower scores indicating a higher likelihood of mortality and unfavourable functional recovery. Studies suggest that combining NSE measurements with GCS scores can enhance prognostic accuracy, providing a comprehensive assessment of a patient's neurological status and potential recovery trajectory, thereby guiding treatment decisions effectively in critical care settings. We posited that the NSE-to-GCS ratio might provide an improved evaluative measure for the prognosis of patients with severe TBI, thereby furnishing a reference for refining patient prognostication.

## Materials and methods

### Study population

Between July 2020 and June 2023, we selected 63 unconscious patients with severe cranio-cerebral trauma who were transferred to the neonatal intensive care unit (NICU) for hospitalisation following emergency cranio-cerebral surgery. The cohort consisted of 52 males and 11 females, with an average age of 52.3±14.1 years. Among these patients, 23 cases presented intracerebral hematoma, 47 cases had dural hematoma, 4 cases exhibited diffuse axonal injury, 21 cases showed cerebral hernia, 54 cases displayed subarachnoid haemorrhage, 53 cases suffered from fractures, 19 cases had hypertension, 7 cases had diabetes, and 3 cases had heart disease. Furthermore, 40 cases were linked to traffic accidents, 12 cases to falls, and 11 cases to other fall-related injuries.

Since patients with cerebral haemorrhage admitted to the NICU were deemed critically ill to provide effective consent, authorisation was sought from first-degree relatives, as permitted by law.

### Selection of participants

Inclusion criteria were as follows: (1) Meeting diagnostic standards for severe traumatic brain injury; (2) Age ≥18 years with head injuries caused by accidents, confirmed as traumatic brain injuries by head CT, with less than 24 hours from injury to admission, and all patients undergoing emergency cranio-cerebral surgery; (3) GCS score 10; (4) Expected survival time ≥7 days. Exclusion criteria were as follows: (1) Patients with severe hemodynamic instability, estimated to have a survival time of less than 7 days; (2) Patients with significant diseases such as stroke, malignant tumors, cirrhosis, uremia, heart failure, AIDS, or active sexually transmitted and immune system diseases; (3) Patients with prolonged use of hormones and immunosuppressants; (4) Patients with severe infections like COVID-19 within the past 4 weeks; (5) Clinical data incompleteness or loss to follow-up.

### Comprehensive treatment

During hospitalisation, all patients received tailored early nutritional support, neural nutrition, cranial pressure-reducing hydration, anti-infection measures, aerosolised sputum aspiration, maintenance of water and electrolyte balance, and stabilisation of the internal environment. Patients were assisted with ventilators for breathing and proactive prevention of complications. Depending on patients' initial conditions, bedside joint movements and acupuncture were employed for rehabilitation.

### Clinical characteristics

Baseline characteristics of critically injured TBI patients were collected, encompassing age, gender, admission time (in hours) from injury, admission GCS score, admission APACHE II score, complications (such as intracerebral hematoma [ICH], dural hematoma, diffuse axonal injury, hernia, subarachnoid hemorrhage [SAH], fracture), prior medical history (including hypertension, diabetes, heart disease), and 90-day outcomes.

### Parameters

Before admission, blood tests were performed, including absolute neutrophil count, absolute lymphocyte count, and platelet count. The neutrophil-to-lymphocyte ratio (NLR), platelet-to-lymphocyte ratio (PLR), and systemic immune inflammation index (SII) were calculated (NLR=neutrophil count/lymphocyte count, PLR=platelet count/lymphocyte count, SII =neutrophil countXplatelet count/lymphocyte count/1000). IL-6, PCT, and DD levels were recorded before admission. Unfortunately, preoperative NSE detection was unsuccessful.

After comprehensive treatment, on the seventh day following admission, we assessed NSE, blood osmolality, blood routine (including calculated NLR, PLR, and SII), IL-6, PCT, and DD, and re-evaluated GCS levels. NGR7 (NSE7/GCS7) was calculated. The laboratory department of Baoding First Central Hospital analysed all these indicators.

### Assessment of neurological function (GCS)

The Glasgow Coma Scale (GCS) comprises three standardised scoring components: eye response (up to 4 points), best verbal response (up to 5 points), and best motor response (up to 6 points). These scores are summed for a total score ranging from 3 to 15. A score of 15 signifies clear consciousness; 13-14 points indicate mild coma; 9-12 points suggest moderate coma; and 3-8 points signify severe coma. A lower score corresponds to a more severe impairment in consciousness.

### Outcome assessment

The Glasgow Outcome Scale (GOS) was utilised to evaluate the prognosis of patients 90 days after experiencing severe TBI through telephone follow-up. Based on their GOS scores, the patients were categorised into two groups: a poor prognosis group (scoring 1-3 points) and a good prognosis group (scoring 4-5 points). The GOS employs specific criteria for scoring as follows: A score of 1 signifies death. A score of 2 indicates a vegetative state, characterised by unconsciousness, the presence of a heartbeat and breathing, occasional eye opening, as well as local motor responses such as sucking and yawning. A score of 3 denotes severe disability, where patients are conscious but grapple with substantial cognitive, speech, and physical motor impairments necessitating round-the-clock care. A score of 4 represents moderate disability, characterised by conscious functioning with slight setbacks in cognition, behaviour, and personality. Patients may also experience mild hemiplegia, ataxia, speech difficulties, and other disabilities. Despite these challenges, they maintain a degree of independence in their daily life, family, and social activities. A score of 5 signifies a good recovery, indicating the ability to reintegrate into normal social routines and resume work and school activities, albeit with some mild lingering effects.

### Statistical analysis

Continuous data were presented as mean ± standard deviation (SD) or median with interquartile range. Categorical variables were expressed as counts and/or percentages. The method of analysis depended on variable distribution. Categorical data were analysed using the χ^2^ test or Fisher's exact test. Differences between groups were assessed using one-way ANOVA or independent sample T-test for normally distributed parameters, and χ^2^ tests for between-group comparisons. The Fisher's exact test was used for patient demographic characteristics and outcomes. Binary logistic regression was employed to analyse patient prognosis risk factors. The predictive capabilities of NGR7 and the optimal cut-off value for achieving the best sensitivity and specificity for the 90-day outcome were determined through receiver operating characteristic (ROC) analysis. Pearson correlation analysis was used to show the association between potential risk factors and the 90-day outcome of TBI. Data entry and storage were conducted in Excel, while statistical analysis was performed using Free statistics software versions 1.7 and GraphPad Prism 9.4.1 software. A two-sided p-value <0.05 was considered statistically significant.

## Results

### Enrollment and characteristics of the patients

Following adherence to the inclusion and exclusion criteria, 63 severe TBI patients were chosen for the study. This comprised 26 patients in the good-prognosis group and 37 in the poor-prognosis group. The average age of all patients was 52.3±14.1 years, with an average length of stay in the NICU of 13.9±6.8 days. The good prognosis group had an average age of 47.8±14.7 years and NICU stay of 11.7±6.1 days. In comparison, the poor prognosis group had an average age of 55.5±13.1 years and NICU stay of 15.5±6.8 days. Significant differences existed in age and NICU stay time between the two groups (both p<0.05), as shown in Table 1.

**Table 1:** Comparison of demographic characteristics and clinical parameters between the good prognosis group and the poor prognosis group.

Characteristic	Total (n=63)	Good (n=26)	Poor (n=37)	p
Man, n (%)	52 (82.5)	21 (80.8)	31 (83.8)	0.750
Age, Mean ± SD	52.3±14.1	47.8±14.7	55.5±13.1	0.034*
Time of NICU stay, Mean ± SD	13.9±6.8	11.7±6.1	15.5±6.8	0.027*
Reason, n (%)				0.490
Traffic accident	40 (63.5)	18 (69.2)	22 (59.5)	
High drop injury	11 (17.5)	5 (19.2)	6 (16.2)	
Fall injury	12 (19.0)	3 (11.5)	9 (24.3)	
Time from injury to admission, Mean ± SD	5.0±2.7	5.0±2.4	4.9±2.9	0.916
Admission GCS, Mean ± SD	4.8±2.1	5.2±2.0	4.4±2.0	0.130
APACHEII, Mean ± SD	19.3±5.7	18.0±5.8	20.2±5.5	0.138
Hernia, n (%)	21 (33.3)	6 (23.1)	15 (40.5)	0.148
Fracture, n (%)	53 (84.1)	23 (88.5)	30 (81.1)	0.504
SAH, n (%)	54 (85.7)	20 (76.9)	34 (91.9)	0.144
ICH, n (%)	54 (85.7)	20 (76.9)	34 (91.9)	0.428
DAI, n (%)	4 (6.3)	1 (3.8)	3 (8.1)	0.637
DH, n (%)	47 (74.6)	19 (73.1)	28 (75.7)	0.816
Hypertension, n (%)	19 (30.2)	8 (30.8)	11 (29.7)	0.929
Diabetes, n (%)	7 (11.1)	4 (15.4)	3 (8.1)	0.434
Heart disease, n (%)	3 (4.8)	0 (0)	3 (8.1)	0.261

### Comparison of the inflammatory markers between the two groups

When comparing the inflammatory markers between the groups, the poor prognosis group exhibited significantly higher NLR and IL-6 levels before admission compared to the good prognosis group (p<0.05). However, no statistically significant differences were noted in PLR, SI I, PCT, and DD levels, both before admission and on the seventh day after admission (p>0.05), as outlined in Table 2.

**Table 2:** Comparison of the perioperative inflammatory markers between the two groups (Mean ± SD). Abbreviations: NLR, neutrophil absolute counts/lymphocyte absolute counts; PLR, platelet count/lymphocyte absolute counts; SII, neutrophil absolute countsXplatelet count/lymphocyte absolute counts /1000; IL-6, interleukin 6; PCT, procalcitonin; DD, D-dimer; D0, before admission; D7, on seventh day of admission; *p<0.05.

Marker	Time	Good (n = 26)	Poor (n = 37)	p	t
NLR	D0	7.8±4.0	15.2±11.9	0.003*	9.377
	D7	6.2±4.1	7.4±4.2	0.301	1.086
PLR	D0	132.6±86.4	188.0±145.8	0.088	3.004
	D7	158.9±69.7	135.7±70.2	0.201	1.675
SII	D0	2.1±1.8	2.7±1.9	0.234	1.446
	D7	1.3±0.7	1.1±0.6	0.253	1.331
PCT (ng/mL)	D0	2.4±2.8	2.8±3.3	0.602	0.275
	D7	1.4±1.6	1.0±1.4	0.341	0.923
IL-6 (pg/mL)	D0	63.5±42.0	138.8±144.8	0.013*	6.623
	D7	36.2±30.4	53.7±75.9	0.269	1.243
DD (mg/L)	D0	30.8±23.4	23.1±24.9	0.221	1.532
	D7	6.2±4.6	6.5±8.3	0.880	0.023

### Perioperative neurological function assessment in both groups

On the seventh day of admission, the GCS score (GCS7) in the poor prognosis group was markedly lower than that in the good prognosis group (p<0.05). Additionally, NGR and NSE levels on the seventh day of admission were significantly higher in the poor-prognosis group than in the good-prognosis group, with statistically significant differences (p<0.05). These findings are illustrated in Table 3.

**Table 3:** Comparison of D7 neurological function assessment between the two groups (Mean ± SD).

variable	Time	Good<br>(n = 26)	Poor<br>(n = 37)	p	t
GCS7	D7	8.1±2.0	5.7±2.6	<0.001*	15.806
NGR7	D7	3.0±1.6	14.9±11.2	<0.001*	28.768
NSE7	D7	23.9±15.2	69.0±44.4	<0.001*	24.763
OSM7	D7	299.3±10.4	302.4±14.6	0.354	0.874

### The risk factors of 90-day prognosis

**Figure 1:**
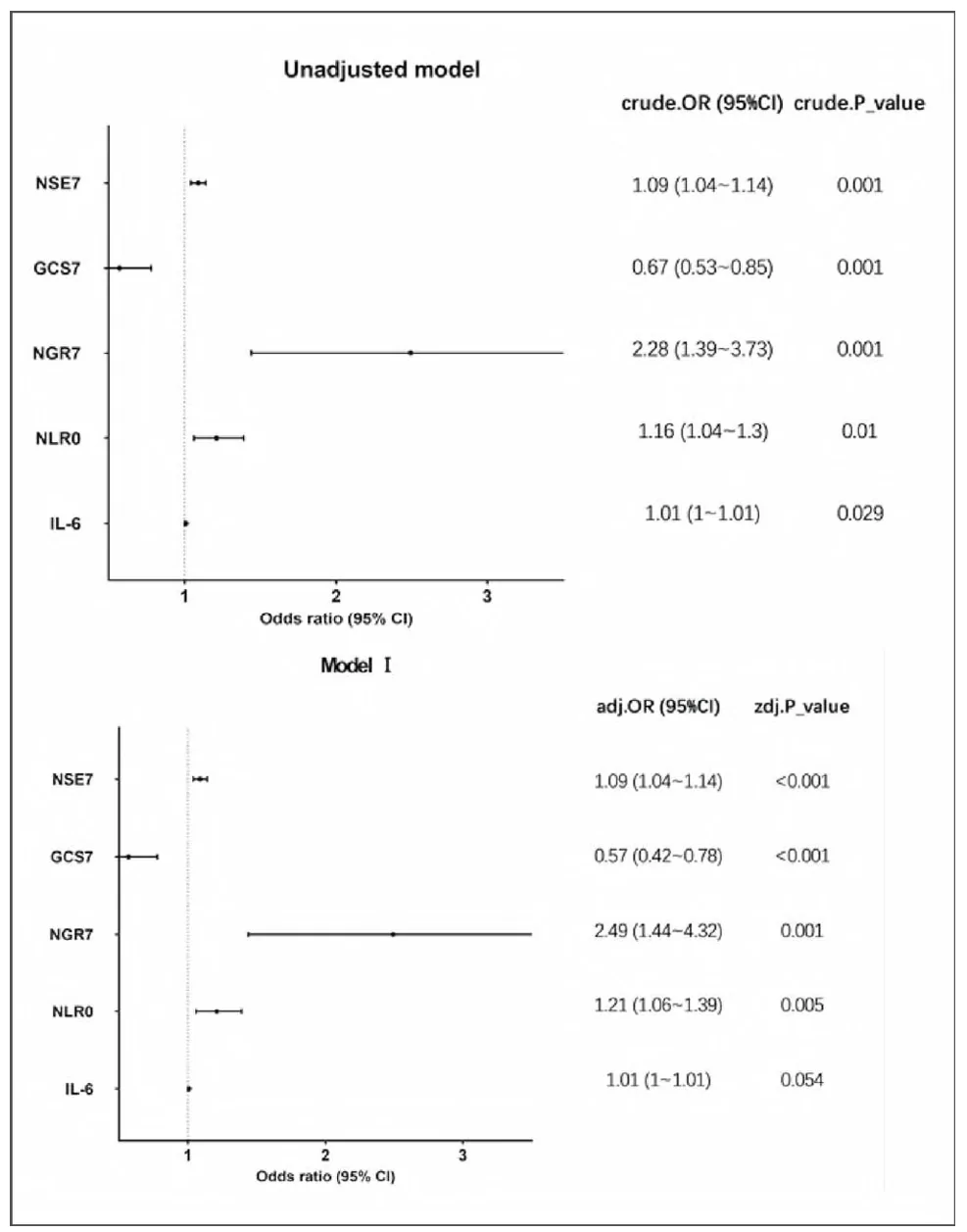
The binary logistic hierarchical analysis of the association between different parameters and TBI with poor prognosis. a) Model II, Model I + GCS + ICH + SAH + DAI + DH; b) Model III, Model I + Hypertension + Diabetes + heart disease

**Figure 2:**
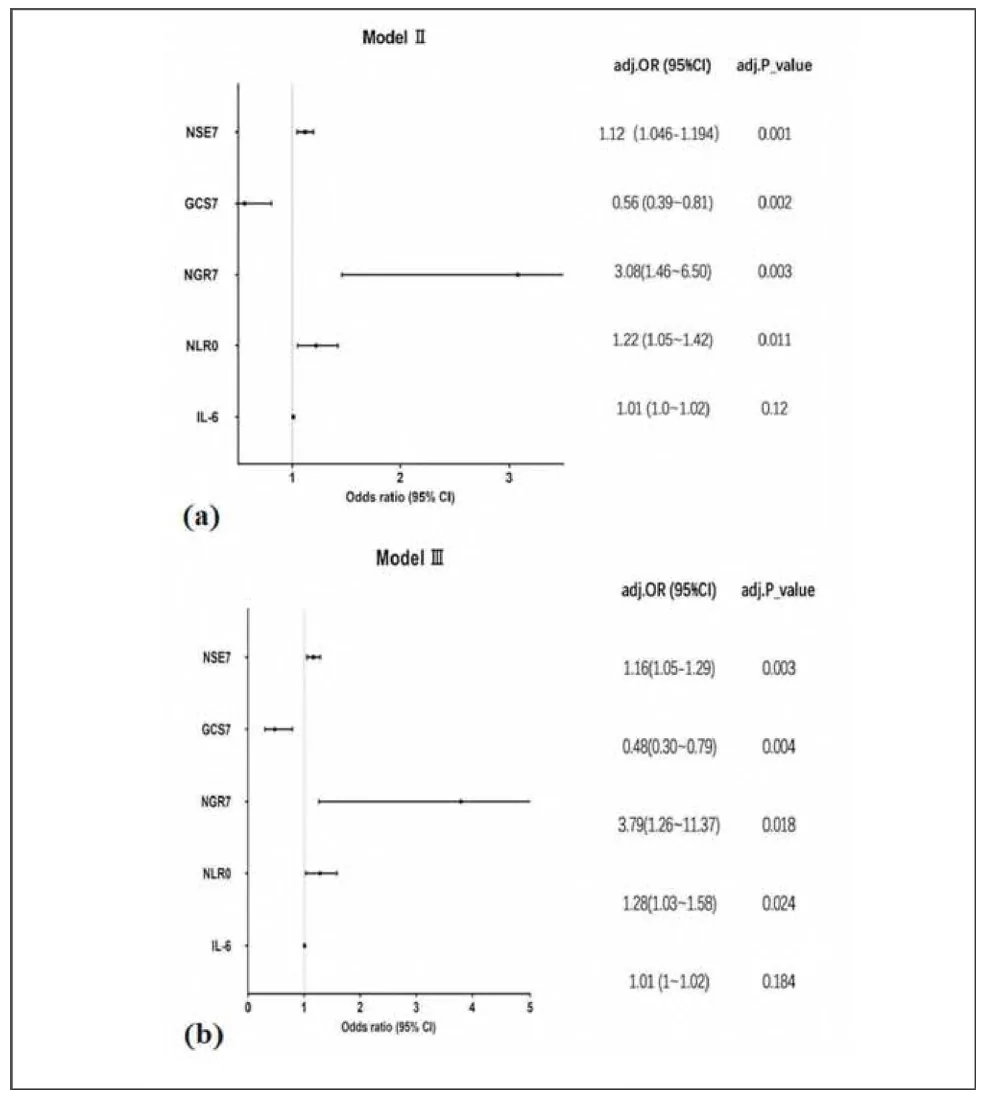
The binary logistic hierarchical analysis of the association between different parameters and TBI with poor prognosis. a) Model II, Model I + GCS + ICH + SAH + DAI + DH; b) Model III, Model I + Hypertension + Diabetes + heart disease

Logistic regression analysis across three models indicated that NSE7, GCS7, NGR7, and preadmission NLR0 levels could influence the 90-day prognosis of severe TBI (all p<0.05). Following adjustment for variables including age, sex, and ICU stay length, each 1-unit increase in NGR7 corresponded to a 3.79-fold increase in the risk of poor prognosis for severe TBI (OR=3.79, 95% CI: 1.26-11.37). Similarly, a 1-unit increase in NSE7 was associated with a 16% increased risk of poor prognosis (OR=1.16, 95% CI: 1.05-1.29). Patients with lower GCS7 scores had a 0.48 times higher risk of poor prognosis than those with higher GCS scores on the same day (OR=0.48, 95%CI: 0.30-0.79). Furthermore, each 1-unit increase in NLR before admission was associated with a 28% increased risk of poor prognosis (OR=1.28, 95% CI: 1.03-1.58), as depicted in Figure 1 and Figure 2.

### Value analysis of risk factors

**Figure 3:**
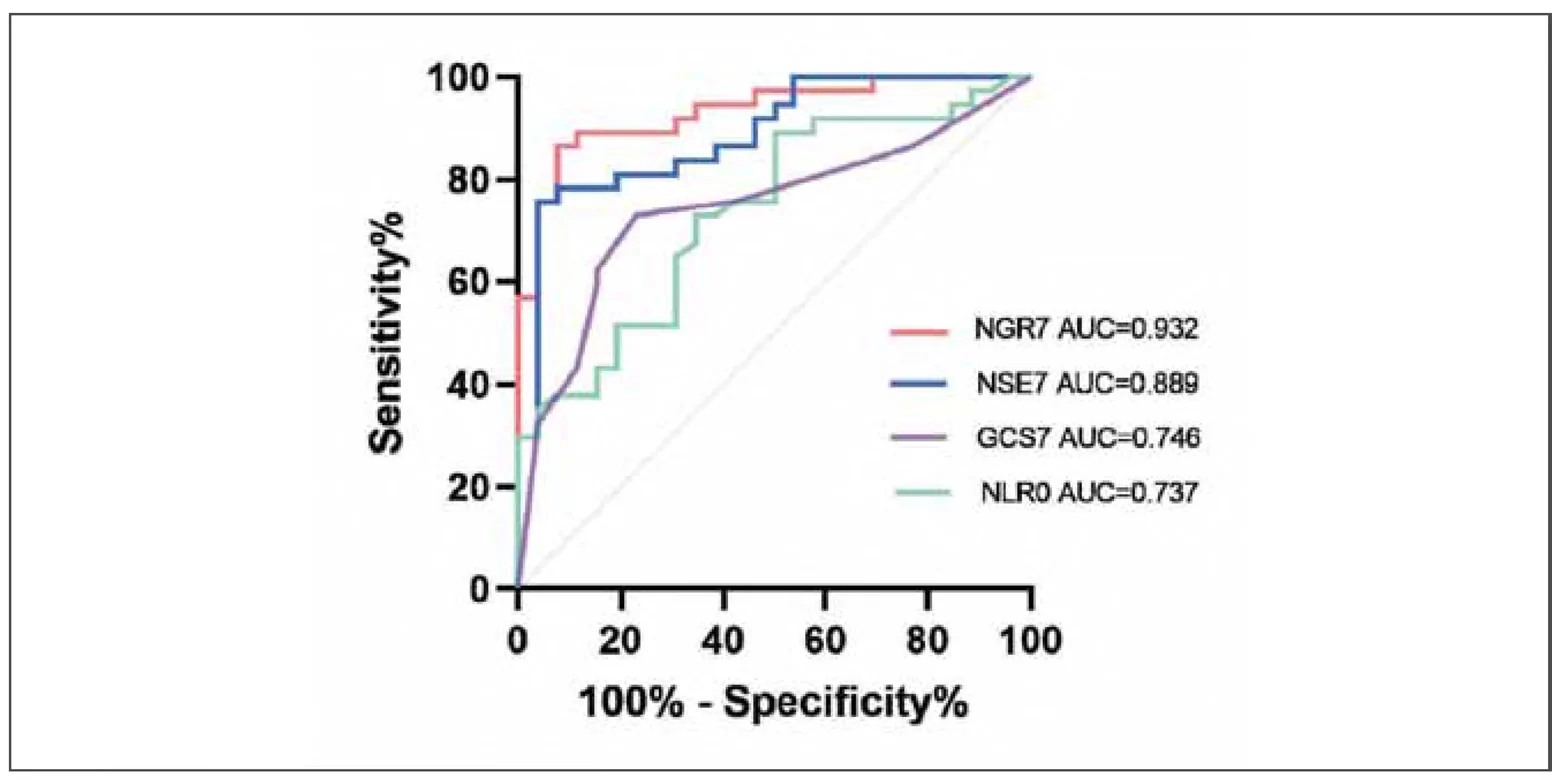
ROC curve of NLR0, NSE7, GCS7 and NGR7.

Based on the results of logistic regression analysis, NLR0, NSE7, GCS7, and NGR7 underwent ROC curve analysis. The outcomes revealed that the area under the curve (AUC) for NLR0 was 0.737 (95%CI=0.614-0.860), with an optimal cut-off value of 5.95, sensitivity of 89.19%, and specificity of 50.0%. Similarly, the AUC for NSE7 was 0.889 (95%CI=0.808-0.970), with an optimal cut-off value of 38.67, sensitivity of 75.68%, and specificity of 96.15%. The AUC for GCS7 was 0.746 (95%CI=0.622-0.870), with an optimal cut-off value of 7.5, sensitivity of 72.97%, and specificity of 76.92%. Finally, the AUC for NGR7 was 0.9з2 (95%CI=0.872-0.993), with an optimal cut-off value of 4.69, sensitivity of 86.49%, and specificity of 92.31% (as shown in Table 4). Notably, NGR7 exhibited a superior predictive value for the 90-day prognosis of severe TBI compared to other indicators (Figure 3).

**Table 4:** Comparison of D7 neurological function assessment between the two groups (Mean ± SD).

Variable	AUC	cut-off value	Sensitivity %	specificity %	Ybuden index	95%CI	SD	p
NLR0	0.737	5.95	89.19	50	0.320	0.614-0.860	0.063	0.0015
NSE7	0.889	38.67	75.68	96.15	0.718	0.808-0.970	0.041	<0.0001
GCS7	0.746	7.5	72.97	76.92	0.499	0.622-0.870	0.063	0.001
NGR7	0.932	4.69	86.49	92.31	0.788	0.872-0.993	0.031	<0.0001

### Correlation of the 90-day prognosis with risk factors

Pearson correlation analysis demonstrated that the 90-day prognosis of severe TBI patients displayed negative correlations with NLR0, NSE7, and NGR7, and a positive correlation with GCS7 (all p<0.05), as outlined in Table 5.

**Table 5:** Correlation of the 90-day prognosis with risk factors.

Variable	r	P
NLR0	-0.378	0.0031
NSE7	-0.740	<0.0001
GCS7	0.331	0.008
NGR7	-0.702	<0.0001

## Discussion

Severe traumatic brain injury (TBI) leads to high rates of both disability and mortality, placing substantial burdens on both patient families and society. The primary damage resulting from TBI is irreversible, yet secondary injuries might offer potential for prevention or reversal. Hence, secondary injury presents an opportunity for effective TBI treatment. In our investigation, we noted that TBI patients with unfavourable outcomes at the 90-day mark tend to be older and have more extended stays in the neonatal intensive care unit (NICU). Notably, Kornblith ES discovered that in comparison to younger individuals experiencing similar degrees of traumatic brain injury, older individuals face a more gradual recovery process and display poorer neurological, cognitive, and psychosocial outcomes post-injury [3]. Moreover, older individuals exhibit elevated incidence and mortality rates [4]. An examination of 60,000 TBI patients revealed that survivors of TBI had a greater likelihood of transitioning to nursing homes than elderly individuals with non-traumatic brain injuries. The presence of comorbidities in elderly TBI patients places a heightened burden, potentially impeding rehabilitation. Hence, elderly TBI survivors necessitate intensified intervention and treatment within the community [5].

Various biomarkers are believed to play a role in the occurrence of secondary injury following severe TBI. NSE is localised in the cytoplasm of neurons, red blood cells, platelets, and neuroendocrine cells. Its primary function involves participating in the glycolysis processes of neurons and erythrocytes (red blood cells). NSE serves as an indicator of neuronal damage [6][7]. Research has indicated that TBI patients with a GCS score below 7 exhibit notably higher serum NSE levels compared to patients with a GCS score of 7 or above, and a substantial negative correlation exists between GCS scores and NSE levels [8][9]. Moreover, in cases of patients who passed away within 30 days after trauma and had a GCS score of 8 or lower upon admission, serum NSE levels showed marked elevation. Furthermore, heightened cerebrospinal fluid NSE levels have been linked to intracranial hypertension and cerebral perfusion insufficiency [10]. This suggests that NSE levels in peripheral blood and cerebrospinal fluid serve as indicators for assessing TBI severity and predicting unfavourable outcomes.

A recently published study suggested that the optimal cut-off value for the serum NSE levels to admission GCS score ratio (NGR) is 4.25. This ratio demonstrated a sensitivity of 90.91% and specificity of 85.71%, establishing it as an independent predictor of diffuse axonal injury DAI in patients with moderate to severe TBI [11]. Our study observed that among severe TBI patients with unfavourable outcomes at 90 days, those with lower GCS7 scores and higher NGR7 and NSE7 levels on the seventh day of comprehensive treatment exhibited these associations consistently across various adjusted models. ROC curve analysis further revealed that GCS7 7.5, NSE7≥8.67, and NGR7≥4.69 on the seventh day were all associated with poor outcomes at 90 days. Specifically, GCS7 displayed a negative correlation with poor 90-day outcomes, while NSE7 and NGR7 exhibited positive correlations. We propose two potential reasons for the influence of NSE on the seventh day after treatment on prognosis. Firstly, reperfusion brain injury might prompt the release of neuronal NSE, followed by the disruption of the blood-brain barrier due to immune reactions. Secondly, the elevated NSE levels could be attributed to severe neuronal damage during secondary brain swelling and injury [12]. Enhancing cerebral perfusion and minimising reperfusion injury could lead to reduced NSE levels, consequently improving patient prognosis.

Throughout the occurrence and progression of secondary injury in TBI, the release of damage-associated molecular patterns initiates the activation of astrocytes and microglia, hastening the generation of neuroinflammation in the affected area [13]. This process culminates in the release of both pro-inflammatory and anti-inflammatory mediators, along with various growth factors, compromising the integrity of the blood-brain barrier. This disruption triggers local neuroinflammation and systemic inflammatory responses, ultimately resulting in secondary brain damage and neurodegeneration. NLR serves as a straightforward and cost-effective marker of inflammatory response. Demonstrated to be sensitive to the clinical severity of inflammation-related tissue damage, elevated NLR values have been associated with poor prognosis in TBI patients [14]. Interleukin-6 (IL-6) plays a multifaceted role in inflammation, immunity, neurodevelopment, tissue remodelling, blood and vascular function, and metabolism [15].

During the acute phase of brain injury, elevated levels of IL-6 can be detected in cerebrospinal fluid, blood, and brain tissue, reflecting the response to TBI damage [16]. Among TBI patients, elevated IL-6 levels are notably linked with poor prognosis [15][17]. An IL-6 level exceeding 100 pg/mL within 24 hours after TBI is indicative of severe brain injury [18]. However, IL-6's role in the injury response is two-sided. At the same time, it supports neurogenesis and wound healing in animal models of traumatic brain injury; it can also contribute to blood-brain barrier disruption and the progression of brain oedema [15]. In our study, we compared inflammatory markers before and after admission, finding that preadmission NLR and IL-6 levels were associated with poor 90-day outcomes in severe TBI patients. However, through logistic regression analysis using multiple models, we established that only preadmission NLR levels could influence the 90-day prognosis of patients with severe TBI.

Despite the progress made in medical science and the evolution of neuro-monitoring systems, TBI remains a significant contributor to global disability and mortality. Therapeutic studies targeting neurotrauma biomarkers remain relatively scarce. Notably, reports suggest that TBI patients treated with amantadine exhibit a substantial reduction in serum NSE levels by the 7th day of treatment. Additionally, their GCS scores improve significantly as early as the 3rd day of treatment, indicating a potential neuroprotective effect of amantadine [19]. Research also indicates that the early administration of 7-nitroindazole (7-NI) results in a significant decrease in serum NSE levels following TBI. The neuroprotective benefits of 7-NI may stem from its ability to lower nitric oxide content, mitigate brain oedema, reduce blood-brain barrier permeability, and alleviate oxidative stress. Notably, serum NSE levels can function as an indicator of the therapeutic effectiveness of 7-NI [20].

Additionally, the application of doxycycline has shown potential in lowering serum NSE levels in TBI patients while simultaneously improving GCS scores. The early adjunctive use of doxycycline emerges as a promising strategy to mitigate ongoing secondary brain injury in TBI patients [21]. In the realm of inflammation and neurotrauma markers, the future calls for extensive clinical trials to develop novel drugs targeting secondary injury in cranial and brain trauma.

## Conclusion

To summarise, preadmission NLR levels (NLR0), along with post-admission 7th-day NSE levels (NSE7), GCS scores (GCS7), and NGR7, all serve as independent predictive factors for evaluating the 90-day prognosis of TBI. In our study, NGR7 emerged as the most potent predictor for 90-day outcomes in severe TBI patients, exhibiting an AUC of 0.932 and an optimal threshold value of 4.69. We recommend assessing GCS scores on the 7th day of comprehensive treatment, combined with concurrent NSE measurement and NGR calculation, to evaluate the 90-day prognosis of traumatic brain injury patients in the neurointensive care unit (NICU). This approach holds clinical promise and can serve as an additional biomarker in practical clinical settings to identify high-risk patients with unfavourable short-term outcomes, including mortality. However, further comprehensive large-scale studies are necessary to validate this significant finding.

## Dodatak

### Limitations

In summary, our study demonstrates that the NSE-to-GCS ratio (NGR) on the seventh day post-admission serves as a significant independent predictor of 90-day outcomes in patients with severe TBI. The findings indicate that NGR not only correlates strongly with patient prognosis but also exhibits superior diagnostic accuracy compared to other established markers, with an area under the curve (AUC) of 0.932. This highlights its potential utility as a clinical tool for assessing the risk of unfavourable outcomes. The incorporation of NGR into clinical practice could enhance decision-making processes in neurointensive care units by providing a more nuanced understanding of patient prognosis. By combining objective measures of neuronal damage, as represented by NSE, with the clinical assessment of the consciousness supplied by GCS, healthcare providers can better identify high-risk patients who may require more intensive monitoring and intervention. Furthermore, utilising NGR as a predictive tool could facilitate early interventions aimed at mitigating secondary injuries, ultimately improving patient outcomes. As such, we advocate for the integration of NGR into routine prognostic assessments for severe TBI patients, alongside ongoing research to validate its effectiveness across larger cohorts and diverse clinical settings.

### Ethical approval

Our case study adheres to the principles outlined in the Declaration of First Central Hospital of Baoding. Consent from the patients' family members has been secured. The Ethical Committee of Baoding First Central Hospital, Hebei, approved the study protocols and treatment procedures involved (approval no. [2021] 034).

### Authors' contributions

Chunying Zhu and Xinjun Shan designed this study and prepared the manuscript, and are responsible for its accuracy and completeness, with equal overall contributions. Wei Li collected and analysed clinical data. Yingfu Zhang and Huan Wang extracted the data and significantly revised this manuscript.

### Funding

This study was supported by the Baoding Science and Technology Bureau (Grant No. 2141ZF058).

### Conflict of interest statement

All the authors declare that they have no conflict of interest in this work.
